# Chronic constipation diagnosis and treatment evaluation: the “CHRO.CO.DI.T.E.” study

**DOI:** 10.1186/s12876-016-0556-7

**Published:** 2017-01-14

**Authors:** Massimo Bellini, Paolo Usai-Satta, Antonio Bove, Renato Bocchini, Francesca Galeazzi, Edda Battaglia, Pietro Alduini, Elisabetta Buscarini, Gabrio Bassotti, ChroCoDiTE Study Group, AIGO, Massimo Bellini, Paolo Usai Satta, Antonio Bove, Renato Bocchini, Edda Battaglia, Pietro Alduini, Gabrio Bassotti, Antonio Balzano, Piero Portincasa, Leonilde Bonfrate, Lucia D’Alba, Danilo Badiali, Santino Marchi, Dario Gambaccini, Maria Cristina Neri, Nicola Muscatiello, Michele Di Stefano, Claudio Giannelli, Fabio Goffredo, Luigi Turco, Salvatore Camilleri, Giovanni Ceccarelli, Paola Iovino, Luigi Maria Montalbano, Gaetano Morreale, Silvia Rentini, Vincenzo Savarino, Sergio Segato, Elisabetta Buscarini, Guido Manfredi, Renato Cannizzaro, Sandro Passaretti, Matteo Alessandri, Federico Corti, Rosario Cuomo, Francesco Paolo Zito, Carmine Mellone, Roberta Barbera, Giuseppe Milazzo, Filippo Pucciani, Marco Soncini, Maria Antonia Lai, Maurizio Ruggeri, Maria Flavia Savarese, Manuela De Bona, Elisabetta Surrenti, Andrea Arini, Marco Dinelli, Gioacchino Leandro, Sergio Peralta, Raffaele Manta, Mariano Quartini, Francesco Torresan, Luigi Vilardo, Antonino Pulvirenti D’Urso, Ottaviano Tarantino, Roberto Antonio Noris, Fabio Monica, Maurizio Carrara, Alessandra Losco, Adriano Lauri, Matteo Neri, Mario Grassini

**Affiliations:** 1 Gastrointestinal Unit, Department of Gastroenterology, University of Pisa, Via Paradisa, 2, 56127 Pisa, Italy; 2 Brotzu Hospital, Gastroenterology Unit, Cagliari, Italy; 3 AORN “A. Cardarelli”, Naples, Italy; 4 Malatesta Novello, Private Hospital, Cesena, Italy; 5 Division of Gastroenterology, Department of Surgery, Oncology and Gastroenterology, University of Padua, Padua, Italy; 6 Cardinal Massaia Hospital, Gastroenterology Unit, Asti, Italy; 7 San Luca Hospital, Digestive Endoscopy Unit, Lucca, Italy; 8 Gastroenterology and Digestive Endoscopy Unit, Maggiore Hospital, Crema, Italy; 9 Gastrointestinal and Hepatology Section, Clinical & Experimental Medicine, Perugia, Italy

**Keywords:** Functional constipation, Irritable bowel syndrome, Diagnosis, Treatment

## Abstract

**Background:**

According to Rome criteria, chronic constipation (CC) includes functional constipation (FC) and irritable bowel syndrome with constipation (IBS-C). Some patients do not meet these criteria (No Rome Constipation, NRC). The aim of the study was is to evaluate the various clinical presentation and management of FC, IBS-C and NRC in Italy.

**Methods:**

During a 2-month period, 52 Italian gastroenterologists recorded clinical data of FC, IBS-C and NRC patients, using Bristol scale, PAC-SYM and PAC-QoL questionnaires. In addition, gastroenterologists were also asked to record whether the patients were clinically assessed for CC for the first time or were in follow up. Diagnostic tests and prescribed therapies were also recorded.

**Results:**

Eight hundred seventy-eight consecutive CC patients (706 F) were enrolled (FC 62.5%, IBS-C 31.3%, NRC 6.2%). PAC-SYM and PAC-QoL scores were higher in IBS-C than in FC and NRC. 49.5% were at their first gastroenterological evaluation for CC. In 48.5% CC duration was longer than 10 years. A specialist consultation was requested in 31.6%, more frequently in IBS-C than in NRC. Digital rectal examination was performed in only 56.4%. Diagnostic tests were prescribed to 80.0%. Faecal calprotectin, thyroid tests, celiac serology, breath tests were more frequently suggested in IBS-C and anorectal manometry in FC. More than 90% had at least one treatment suggested on chronic constipation, most frequently dietary changes, macrogol and fibers. Antispasmodics and psychotherapy were more frequently prescribed in IBS-C, prucalopride and pelvic floor rehabilitation in FC.

**Conclusions:**

Patients with IBS-C reported more severe symptoms and worse quality of life than FC and NRC. Digital rectal examination was often not performed but at least one diagnostic test was prescribed to most patients. Colonoscopy and blood tests were the “first line” diagnostic tools. Macrogol was the most prescribed laxative, and prucalopride and pelvic floor rehabilitation represented a “second line” approach. Diagnostic tests and prescribed therapies increased by increasing CC severity.

**Electronic supplementary material:**

The online version of this article (doi:10.1186/s12876-016-0556-7) contains supplementary material, which is available to authorized users.

## Background

Chronic constipation (CC) is a common and extremely troublesome disorder that has a negative impact on social and professional life, reduces the quality of life (QoL) and represents a heavy economic burden [1–5]. CC affects about 12–17% of the world population, with a higher prevalence among females and elderly people [6–9].

A considerable amount (16 to 40%) of CC patients in different countries use laxatives, and their use is related to increasing age, symptom frequency and duration of constipation; in the USA more than $800 million are spent on laxatives each year [10, 11].

The most widely used criteria to assess CC are the Rome Criteria [12] (Table 1) which separate constipation in functional constipation (FC) and irritable bowel syndrome with constipation (IBS-C). The presence of abdominal pain relieved by defecation characterizes IBS-C. Moreover, some patients consider themselves constipated even when not showing signs or symptoms consistent with Rome criteria (here defined as “No-Rome Constipation”, NRC) [13].

**Table 1:** Rome III criteria for functional constipation and irritable bowel syndrome

Functional Constipation
* Diagnostic criteria* ^a^
1. Must include two or more of the following:
α. Straining during at least 25% of defecations
β. Lumpy or hard stools in at least 25% of defecations
γ. Sensation of incomplete evacuation for at least 25% of defecations
δ. Sensation of anorectal obstruction/blockage for at least 25% of defecations
ε. Manual manoeuvres to facilitate at least 25% of defecations (e.g. digital evacuation, support of the pelvic floor)
η. Fewer than three defecations per week
2. Loose stools are rarely present without the use of laxatives
3. Insufficient criteria for irritable bowel syndrome
^a^Criteria fulfilled for the last 3 months with symptoms onset at least 6 months prior to diagnosis
Irritable Bowel Syndrome with Constipation
*Diagnostic criteria* ^*a*^
Recurrent abdominal pain or discomfort ^b^ at least 3 days/month in the last 3 months associated with two or more of the following:
-Improvement with defecation
-Onset associated with a change in frequency of stool
-Onset associated with a change in form (appearance) of stool (hard or lumpy stools ≥25% and loose or watery stools <25% of bowel movements)

^a^Criteria fulfilled for the last 3 months with symptoms onset at least 6 months prior to diagnosis

^b^ “Discomfort” means an uncomfortable sensation not described as pain

At present it is unclear whether gastroenterologists use the same diagnostic and therapeutic approach in these different groups of patients.

### Objective of the study

#### Primary endpoints

- To describe the diagnostic tools used and the treatments suggested by Italian gastroenterologists for CC patients.

#### Secondary endpoints

- To assess, among CC patients, the distribution of FC, IBS-C and NRC and the severity of symptoms and QoL.
- To evaluate whether the diagnosis of FC, IBS-C and NRC could affect the use of the diagnostic tools and the choice of the therapy.
- To evaluate other possible potential factors affecting the use of the diagnostic tools and the therapeutic choices in CC patients.

## Methods

### Study population and questionnaires

Fifty two gastroenterologists belonging to different gastroenterological units in Italy on behalf of the Italian Association of Hospital Gastroenterologists and Endoscopists (AIGO), recorded clinical and demographic data of all patients consecutively referred for CC in a two month period (September-October 2013).

Bristol scale [14] was used to assess the stool consistency in the previous three months, while symptoms were classified according to Rome III criteria in order to verify whether the patients could be diagnosed as FC, IBS-C, or NRC. In addition, gastroenterologists were also asked to record whether the patients were clinically assessed for CC for the first time or were in follow up. Diagnostic tests, recommended specialist consultations and prescribed therapies were also recorded.

Furthermore, patients were required to fill the Patient Assessment of Constipation-Symptoms (PAC-SYM) and the Patient Assessment of Constipation-Quality of Life (PAC-QoL) questionnaires.

PAC-SYM is a 12-item self-reported questionnaire developed to assess the frequency and severity of CC symptoms. It is divided into three symptom subscales: abdominal (items 1–4), rectal (items 5–7), and stool (items 8–12) [15].

PAC-QoL is a 28 item self-reported questionnaire used to measure the patient’s QoL. It is divided into four subscales: physical discomfort (items 1–4), psychosocial discomfort (items 5–12), worries and concerns (items 12–23), and satisfaction (items 24–28) [16].

For both questionnaires, items are scored on a five-point Likert scale (0–4), with 4 indicating the worst symptom severity.

#### Inclusion criteria

-Patients aged over 18 years evaluated for CC.

#### Exclusion criteria

-Presence of known or suspected severe organic disease potentially causing constipation and/or psychiatric disease potentially interfering with questionnaires compilation.

-Patients assuming potentially constipating drugs or the onset of constipation after starting any kind of drug.

#### Statistical analysis

Data were analyzed by means of the SAS® System for Windows, version 9.2.

A prevalence approach was adopted and no imputation was performed for any missing data.

The association between categorical variables was analyzed using Chi-Square test or Fisher’s exact test (for cell frequencies < 5). In order to correct for multiple comparisons, pairwise tests were adjusted using the Bonferroni method.

The association between a continuous and a categorical variable (with two categories) was analyzed by the Wilcoxon-Mann–Whitney test. Finally, the association between a continuous and a categorical variable was analyzed by the Kruskal-Wallis test (or by the ANOVA in case of normal distribution). In case of pairwise comparisons, the Dunn’s test was performed. The correlation between two continuous variables was summarized by the Pearson’s correlation coefficient in case of normal data distribution, or by the Spearman’s correlation coefficient otherwise.

All statistical tests were performed with a two-sided significance level α = 0.05, therefore *p*-values lower than 0.05 were considered statistically significant.

The PAC-SYM and PAC-QoL total and domain scores were calculated as detailed in Additional file 1, respectively.

PAC-SYM total score and PAC-QoL total score were also analyzed through multivariate regression models, adjusting for the following independent variables: age, sex, diagnosis, duration of CC.

## Results

Data from 878 CC patients (33.9% in Northern Italy, 32.4% in Center Italy and 33.7% in Southern Italy), 706 women (80.4%) and 172 men (19.6%), mean age: 51.0 ± 16.8 years (F 49.6 ± 16.6 years; M 56.9 ± 16.5 years) were obtained. Their body mass index (BMI) was 23.7 ± 4.0 kg/m^2^. Four hundred thirty-five out of 878 patients (49.5%) were at their first gastroenterological evaluation for CC. According to Rome III criteria the patients were classified as FC: 549 (62.5%); IBS-C: 275 (31.3%); NRC: 54 (6.2%).

IBS-C patients were younger (46.9 ± 16.2 years) than FC (52.8 ± 16.6) and NRC (53.1 ± 18.6) (*p* < 0.0001). The gender distribution was significantly different between the three groups (IBS-C: women 234/275 (85.1%); FC: women 433/549 (78.9%); NRC: women 39/54 (72.2%) (*p* < 0.05) .

The duration of CC was “>1–4 years” in 23.1% (IBS-C: 33.0%; FC: 59.1%; NRC: 7.9%), “≥5years” in 21.1% (IBS-C: 27.0%; FC: 65.4%; NRC: 7.6%) and “>10 years” in 48.5% of the patients (IBS-C: 32.2%; FC: 63.9%; NRC: 4.0%). No significant difference was observed between groups but only a trend toward a shorter duration in NRC could be detected.

Bristol 1–2 was reported in 628/878 (71.5%) patients (IBS-C: 208/275, 75.6%; FC:394/549, 71.8%; NRC: 26/54, 48.2%) (IBS-C vs FC: ns; IBS-C vs. NRC: *p* < 0.001; FC vs NRC: *p* < 0.005).

As shown in Table 2, 73.2% of patients reported at least one comorbidity in the previous year: depression and anxiety were more frequent in IBS-C compared to FC (*p* < 0.01) and NRC (*p* < 0.005), as well as dyspepsia (*p* < 0.05 vs. FC and NRC). Gastroesophageal reflux disease was more frequent in IBS-C compared to NRC (*p* < 0.01) and in FC compared to NRC (*p* < 0.05). Hypertension was found more frequently in FC than in IBS-C (*p* < 0.05).

**Table 2:** Prevalence of comorbidities

	IBS-C: 275	FC: 549	NRC: 54	*p*-value^1^
Dyspepsia	128 (46.5%)	200 (36.4%)	14 (25.9%)^#^	<0.005
Depression/anxiety	111 (40.4%)	164 (29.9%)*	9 (16.7%)**	<0.0005
GERD	98 (35.6%)	167 (30.4%)	8 (14.8%)^## ###^	<0.01
Sleep disturbances	87 (31.6%)	141 (25.7%)	13 (24.1%)	ns
Hypertension	45 (16.4%)	135 (24.6%)^§^	11 (20.4%)	<0.05
Urinary disturbances	52 (18.9%)	115 (20.9%)	8 (14.8%)	ns
Thyroid disease	26 (9.4%)	65 (11.8%)	6 (11.1%)	ns
Vaginitis	29 (10.5%)	46 (8.4%)	4 (7.4%)	ns
Dyspareunia	30 (10.9%)	38 (6.9%)	4 (7.4%)	ns
Diabetes	7 (2.5%)	29 (5.3%)	3 (5.6%)	ns
Fibromyalgia	16 (5.8%)	18 (3.3%)	-	ns
Other	36 (13.1)	56 (10.2%)	7 (13%)	ns

*IBS-C* irritable bowel syndrome with constipation, *FC* functional constipation, *NRC* patients do not accomplish Rome III criteria, *GERD* gastroesophageal reflux disease

^1^ *p* values are referred to the differences between IBS-C, FC and NRC groups, in particular

*p < 0.01 vs IBS-C; **p < 0.005 vs IBS-C; #*p* < 0.05 vs IBS-C and FC; ##*p* < 0.01 vs IBS-C

###*p* < 0.05 vs FC; §*p* < 0.05 vs IBS-C; ns: not statistically significant

The results of PAC-SYM are shown in Table 3: IBS-C mean total score was higher than FC and NRC (*p* < 0.0001) ones. The multivariate regression model suggested that the total score of PAC-SYM (mean: 1.6 ± 0.7) was directly related to the duration of constipation (*p* < 0.01), and to younger age (*p* < 0.0001). Abdominal symptoms subscale was significantly higher in IBS-C than in FC (*p* < 0.05) and in NRC (*p* < 0.0001). In particular, a positive association was detected between each of the first four items (discomfort, pain, bloating and stomach cramps) which constitutes the abdominal subscale and IBS-C (*p* < 0.0001). Fecal symptoms subscale was significantly higher in FC and IBS-C than NRC (*p* < 0.01). Furthermore, there was a positive correlation of the total PAC-SYM score with the number of diagnostic tests (*p* < 0.0005) and of suggested therapies (*p* < 0.05).

**Table 3:** PAC-SYM total score and abdominal, rectal and faecal symptoms subscales: mean values ± SD in all patients and in IBS-C, FC and NRC subgroups

	ALL PATIENTS	IBS-C	FC	NRC	*p*-value^1^
Total score	1.6 ± 0.70	1.75 ± 0.70*	1.56 ± 0.68	1.31 ± 0.70	*p* < 0.0001
Abdominal symptoms	1.53 ± 0.88	1.91 ± 0.74#§	1.37 ± 0.88	1.19 ± 0.86	*p* < 0.0001
Rectal symptoms	0.88 ± 0.86	0.98 ± 0.92	0.85 ± 0.83	0.70 ± 0.78	ns
Faecal symptoms	2.09 ± 0.91	2.09 ± 0.88	2.13 ± 0.92	1.74 ± 0.88^	*p* < 0.01

*IBS-C* irritable bowel syndrome with constipation, *FC* functional constipation, *NRC* patients do not accomplish Rome III criteria

^1^ *p* values are referred to the differences between IBS-C, FC and NRC groups, in particular * *p* < 0.0001 vs FC and NRC; # *p* < 0.0001 vs NRC; § *p* < 0.05 vs FC; ^ *p* < 0.01 vs IBS-C and FC; ns: not statistically significant

In Table 4 the results of PAC-QoL are shown: IBS–C mean total score was higher than FC and NRC (*p* < 0.001); all the subscales, excluding the satisfaction subscale, were significantly higher in IBS-C and in FC than in NRC. Moreover, the multivariate regression model for the total score of PAC-QoL (mean: 1.8 ± 0.7) shows that this was neither related to gender, nor to age or duration of constipation. There was a statistically significant positive correlation with the number of diagnostic tests (*p* < 0.05), the number of suggested therapies (*p* < 0.0001) and the number of specialist consultations (*p* < 0.005).

**Table 4:** PAC-QoL total score and subscales (mean values ± SD) in all patients and in IBS-C, FC and NRC subgroups

	ALL PATIENTS	IBS-C	FC	NRC	*p*-value^1^
Total score	1.77 ± 0.69	1.97 ± 0.70	1.71 ± 0.68	1.44 ± 0.62*	*p* < 0.001
Physical discomfort	1.85 ± 0.88	2.13 ± 0.82	1.75 ± 0.88	1.42 ± 0.82*	*p* < 0.001
Psychosocial discomfort	1.12 ± 0.83	1.38 ± 0.83	1.02 ± 0.80	0.77 ± 0.73*	*p* < 0.001
Worries and concerns	1.72 ± 0.92	1.92 ± 0.93	1.65 ± 0.90	1.36 ± 0.80*	*p* < 0.001
Satisfaction	2.90 ± 0.71	2.89 ± 0.72	2.92 ± 0.69	2.74 ± 0.81	ns

*IBS-C* irritable bowel syndrome with constipation, *FC* functional constipation, *NRC* patients do not accomplish Rome III criteria

^1^ *p* values are referred to the differences between IBS-C, FC and NRC groups, in particular

* *p* < 0.001 vs IBS-C and FC; ns: not statistically significant

Digital rectal examination (DRE) was performed in 495/878 (56.4%), independently from the patients being at their first evaluation (54.7%) or at a follow up visit (56.6%). No relationship with gender was found (104 M: 61.3%; F 391: 55.2%). Patients in whom a DRE was performed were older (52.6 ± 16.6 years vs. 49.4 ± 16.7; *p* < 0.01), and DRE was more often performed by gastroenterologists aged over 40 years than by younger ones (60.1% vs. 44.6%; *p* < 0.0001).

At least a specialist consultation was requested in 277/878 (31.6%) patients, mostly psychiatric/psychological (11.5%), urological (8.1%) and gynecological (12.3% of the women) (Table 5). In IBS-C psychiatric/psychological and gynaecological consultations were more frequently requested than in NRC (*p* < 0.05).

**Table 5:** Specialist consultations requested by the gastroenterologists after their visit

CONSULTATION	878 pts. (%)	IBS-C (%)	FC (%)	NRC (%)	*p*-value^1^
Psychiatrist/psychologist	101 (11.5)	16.0	10.0	3.7*	<0.01
Gynaecologist^a^	87(12.3)	15.4	11.6	2.6*	<0.05
Urologist	71 (8.1)	8.0	8.4	5.6	ns
Surgeon	66 (7.5)	6.6	7.8	9.3	ns
Physiatrist/Physiotherapist	49 (5.6)	6.2	5.8	-	ns
Dietician	46 (5.2)	4.0	5.8	5.6	ns
Rheumatologist	15 (1.7)	2.6	1.3	1.9	ns
Neurological	15 (1.7)	1.8	1.6	1.9	ns
Other	17 (1.9)	2.6	1.8	-	ns

*IBS-C* irritable bowel syndrome with constipation, *FC* functional constipation, *NRC* patients do not accomplish Rome III criteria

^a^calculated on the women sample visited

^1^ *p* values are referred to the differences between IBS-C, FC and NRC groups, in particular; *vs IBS-C and FC; ns: not statistically significant

Diagnostic tests were requested in 702/878 (80.0%) of the patients. Table 6 shows the different tests requested in the whole sample and in the different diagnosis subgroups (IBS-C, FC and NRC). Fecal calprotectin was more frequently prescribed in IBS-C than in FC and NRC (*p* < 0.0001 and *p* < 0.05, respectively). Thyroid function tests (*p* < 0.05), serology for celiac disease (*p* < 0.005), lactose breath test (*p* < 0.01) and glucose breath test (*p* < 0.05) were more frequently suggested in IBS-C than in FC, whereas in FC anorectal manometry was more frequently prescribed than in IBS-C (*p* < 0.05) and defecography more frequently than in NRC (*p* < 0.05). Abdominal ultrasonography was suggested in 22% of the patients without significant differences among groups.

**Table 6:** Diagnostic test requested by the gastroenterologist after their visit

DIAGNOSTIC TEST	878 pts.(%)	IBS-C (%)	FC (%)	NRC (%)	*p*-value^1^
Routine blood tests	472 (53.8)	58.9	51.6	50.0	ns
Thyroid function tests	400 (45.6)	52.4 #	43.2	35.2	<0.05
Colonoscopy	339 (38.6)	34.6	40.6	38.9	ns
Anorectal manometry	306 (34.9)	29.8 #	38.6	22.2	<0.01
Colonic transit time	228 (26.0)	30.6	24.6	16.7	ns
Abdominal ultrasonography	193 (22.0)	22.6	22.2	16.7	ns
RX Defecography	167 (19.0)	16.4	21.5 °	7.4	<0.05
Faecal blood test	153 (17.4)	14.9	18.8	16.7	ns
Coeliac serology	142 (16.2)	22.6^	13.1	14.8	<0.005
Carcinoembryonic antigen assay	78 (8.9)	6.6	9.8	11.1	ns
Faecal calprotectin	64 (7.3)	15.3 * **	3.8	1.9	<0.0001
Stool culture, test for ova and parasites	62 (7.1)	6.6	8.0	-	ns
Lactose Breath Test	35 (4.0)	7.3 §	2.7	-	<0.005
MR Defecography	33 (3.8)	2.6	4.2	5.6	ns
Trans-anal ultrasound	30 (3.4)	4.7	2.9	1.9	ns
Rectosigmoidoscopy	22 (2.5)	2.9	2.6	-	ns
Virtual colonoscopy	19 (2.2)	1.8	2.4	1.9	ns
Barium Enema	14 (1.6)	1.1	1.8	1.9	ns
Glucose Breath test	12 (1.4)	2.9 #	0.7	-	<0.05
Colonic manometry	12 (1.4)	1.8	1.1	1.9	ns
Anal Sphincter Electromyography	5 (0.6%)	0.7	0.6	-	ns
Other	34 (3.9)	4.7	3.6	1.9	ns

*IBS-C* irritable bowel syndrome with constipation, *FC* functional constipation, *NRC* patients do not accomplish Rome III criteria

^1^ *p* values are referred to the differences between IBS-C, FC and NRC groups, in particular

# *p* < 0.05 vs FC; * *p* < 0.0001 vs FC; ** *p* < 0.05 vs NRC; ^ *p* < 0.005 vs FC; § *p* < 0.01 vs FC; ° *p* < 0.05 vs NRC; ns: not statistically significant

Colonoscopy was suggested more in patients ≥50 years than in those <50 years (52.3% vs. 22.5%; *p* < 0.0001), more in males than in females (51.2% vs. 35.6%; *p* < 0.001) and more often at first evaluation than at follow-up (43.2% vs. 32.8%; *p* < 0.005). Also, routine blood tests (61.2% vs. 46.6%; *p* < 0.0001), thyroid function tests (52.0% vs. 40.2%,; *p* < 0.001), carcinoembryonic antigen (11.3% vs. 6.4%; *p* < 0.05), serology for celiac disease (19.1% vs. 13.2%; *p* < 0.05); and stool culture and test for ova and parasites (9.7% vs. 4.2%; *p* < 0.005) were requested more often at first evaluation than at follow-up. On the contrary defecography (2.5% vs. 5.6%; *p* < 0.05) was suggested less frequently at first visit than at follow-up. Serology for celiac disease was suggested more frequently in patients <50 years old than in patients ≥50 years old (22.0% vs. 11.3%; *p* < 0.0001).

Table 7 shows the suggested therapies, overall and by diagnosis. In 863/878 patients (98.3%) at least one treatment was given. Lifestyle and dietary changes were the most frequent suggestions, whereas macrogol and fiber supplements were largely the most frequently prescribed substances.

**Table 7:** Suggested therapies requested by the gastroenterologist after their visit

THERAPIES	878 pts.(%)	IBS-C (%)	FC (%)	NRC (%)	*p*-value^1^
Life style recommendations	722 (82.2)	84.7	81.8	74.1	ns
Dietary suggestions	749 (85.3)	85.5	85.8	79.6	ns
Fibre supplements	489 (55.7)	60.7	53.2	55.6	ns
Herbal remedies	46 (5.2)	6.2	4.9	3.7	ns
Probiotics	318 (36.2)	40.4	33.5	42.6	ns
Lactulose/lactitole	58 (6.6)	4.7	7.8	3.7	ns
Macrogol	609 (69.4)	71.6	70.9	42.6 #	<0.0001
Saline laxatives	31 (3.5)	4.0	3.5	1.9	ns
Stimulant laxatives	55 (6.3)	6.2	6.7	1.9	ns
Softening laxatives	46 (5.2)	5.1	5.7	1.9	ns
Prucalopride	126 (14.4)	13.1	15.7	7.4	ns
Suppositories/micro-enemas	198 (22.6)	23.6	21.3	29.6	ns
Enemas	238 (27.1)	29.5	26.6	20.4	ns
Antispasmodics	146 (16.6)	27.6 * **	11.7	11.1	<0.0001
Anti-bloating agents	191 (21.8)	29.1 ^	18.6	16.7	<0.005
Intestinal antibiotics	55 (6.3)	7.3	5.5	9.3	ns
Anxiolytics	108 (12.3)	14.6	11.5	9.3	ns
Antidepressants	60 (6.8)	8.0	6.0	9.3	ns
Psychotherapy	35 (4.0)	6.9 §	2.7	1.9	<0.05
Pelvic floor rehabilitation	169 (19.3)	14.6	22.2 °	13.0	<0.05
Sacral neurostimulation	3 (0.3)	//	0.4	1.9	ns
Anorectal surgery	20 (2.3)	1.5	2.7	1.9	ns
Colectomy	1 (0.1)	//	//	1.9	ns
Other	18 (2.1)	2.2	2.2	//	ns

*IBS-C* irritable bowel syndrome with constipation, *FC* functional constipation, *NRC* patients do not accomplish Rome III criteria

^1^ *p* values are referred to the differences between IBS-C, FC and NRC groups, in particular

# *p* < 0.0001 vs IBS-C and NRC; * *p* < 0.0001 vs FC; ** *p* < 0.05 vs NRC; ^ *p* < 0.005 vs FC; § *p* < 0.05 vs FC; ° *p* < 0.05 vs IBS-C; ns: not statistically significant

Macrogol was suggested more frequently in FC (71.6%) and IBS-C (70.9%) than in NRC (42.6%; *p* < 0.0001). A fiber supplements prescription was slightly more frequent in IBS-C, but no significant difference was detected among IBS-C, FC and NRC. In IBS-C antispasmodics were used more frequently compared to FC and NRC (27.6% vs. 11.7% vs. 11.1%; *p* < 0.0001 and *p* > 0.05, respectively). Antibloating agents (29.1% vs. 18.6%; *p* < 0.005) and psychotherapy (6.9% vs. 2.7%; *p* < 0.05) were most frequently prescribed in IBS-C than in FC, whereas pelvic floor rehabilitation was more frequently suggested in FC than in IBS-C (22.2% vs. 14.6%; *p* < 0.05).

Lactulose/lactitole (8.6% vs. 4.3%; *p* < 0.05), suppositories/micro-enemas (26.4% vs. 18.2%; *p* < 0.005), intestinal antibiotics (9.0% vs. 3.0%; *p* < 0.0005), antidepressants (10.0% vs. 3.0%; *p* < 0.0001), anxiolytics (15.9% vs. 8.1; *p* < 0.001) and pelvic floor rehabilitation (22.0% vs. 15.7%; *p* < 0.05) were more frequently suggested in patients ≥50 years than in patients <50 years, whereas antispasmodics were more frequently prescribed in patients <50 years than in patients ≥50 years (20.0% vs. 13.8%; *p* < 0.05).

Enemas and micro-enemas/suppositories were mainly prescribed not on a daily basis but usually every other day or on demand (24.2% and 19.7%, respectively). Lifestyle changes (87.5% vs. 80.9%; *p* < 0.05) and dietary suggestions (91.1% vs. 83.9%; *p* < 0.05) were more frequently prescribed in males than in females, but anorectal surgery only in females (2.8%).

Probiotics were most frequently prescribed at first visit than at follow-up (40.9% vs. 31.5%; *p* < 0.01), whereas prucalopride and pelvic floor rehabilitation were more often prescribed during a follow-up visit than at first evaluation (20.1% vs. 10.6%, *p* < 0.0001; 23.8% vs. 15.4%, *p* < 0.005, respectively).

A mix of suggestions and drugs was used in many patients: in 59.5% lifestyle suggestions, changes in diet and macrogol; in 50.8% lifestyle suggestions, changes in diet and fiber supplementation; in 37.2% changes in diet, fiber supplementation and macrogol; in 37.1% lifestyle suggestions, fiber supplementation and macrogol; in 33.3% lifestyle suggestions, changes in diet and probiotics.

## Discussion

The present study conveys an important educational message for general practitioners, who see the majority of constipated patients, and for other specialists who could visit patients for possible comorbidities: when collecting the patient’s history, the presence of constipation should be accurately searched and treated (if possible). Waiting so many years before sending constipated patients to a gastroenterologist simply means worsening a patient’s symptoms and his/her QoL [4] and increasing the risk to develop important anatomical alterations such as perineal descent, rectocele, rectal intussusceptions, prolapse, enterocele or sigmoidocele, or increase his/her cardiovascular mortality [17].

Rome criteria seemed accurate to identify constipated patients, since only 6.2% showed NRC.

NRC patients were usually older and often male than IBS-C, and reported fewer and less severe symptoms, softer stools and a better QoL than FC and IBS-C. On the other hand, IBS-C patients were younger and more often female, reported more severe symptoms, harder stools and a worse QoL than NRC and FC. Our results show that Rome III criteria identify patients with more severe constipation.

Recently the new Rome IV criteria have been published [18]. No substantial differences have been introduced regarding definition and classification of functional constipation: simply they state that “abdominal pain and/or bloating may be present but are not predominant symptoms (ie, the patient does not meet criteria for IBS)”. Regarding IBS the term discomfort was eliminated and the frequency of abdominal pain became at least 1 day per week instead of 3 days per month. However we think that these changes would not have a significant impact on the results of our study.

PAC-SYM and PAC-QoL questionnaires showed higher scores in IBS-C group than in FC and NRC: PAC-SYM abdominal symptom subscale, PAC-QoL mean total score, physical discomfort, psychosocial discomfort and worries and concerns subscales were found to be higher in IBS-C. This reflects the close association between the first four items of PAC-SYM (abdominal discomfort, abdominal pain, bloating, stomach cramps) and the typical symptoms of IBS. These symptoms are likely responsible for the lower QoL in IBS-C. Thus, the increase in perception of constipation severity increases impairment of the QoL, also increasing request of diagnostic tests and therapies.

Different clinical characteristics, such as type of constipation and comorbidities, may influence the clinical approach of the gastroenterologists; thus, our primary endpoint was to assess the diagnostic tools and treatment suggested by Italian gastroenterologists to their constipated patients, and the impact on the clinical subgroups.

A surprising result, deserving discussion, is that DRE was not performed in more than 40% of the patients, independently from being at first visit or at follow-up. DRE is the simplest and the most immediate method to assess anal tone and to collect information about the pelvic floor conditions and to detect early forms of rectal cancer or benign diseases [19–22]. These data should be carefully taken into account when carrying out educational campaigns on the diagnosis and treatment of CC.

The presence of comorbidities was likely the main reason for the more frequent requested consultations (psychiatric/psychological, urological, gynecological) underlining the need for a stronger collaboration among different specialists for the correct management of CC, possibly creating multidisciplinary teams.

Regarding the attitude towards diagnostic tests, we want to stress that in about four out of five patients gastroenterologists were not so confident on Rome III criteria, and prescribed at least one diagnostic test, more often in patients at first evaluation, mainly blood tests, but also colonoscopy (requested more frequently in patients older than 50 years), anorectal manometry and measurement of colonic transit time. As already shown in previous studies in a general practitioner setting, abdominal ultrasound, although not recommended by current guidelines, was quite frequently requested, especially when abdominal pain is present [23–26].

To exclude conditions potentially mimicking IBS, laboratory and breath tests were more frequently requested in these patients, whereas in FC, defecography and anorectal manometry were more frequently requested to evaluate the presence of dyssynergic defecation. In NRC patients fewer diagnostic tests were overall required, probably due to less severe symptoms and lesser impairment of the QoL.

Overall, dietetic and lifestyle suggestions were the most frequently suggested therapeutic options (>90% of the patients) (Table 7). However, in the present study, the gastroenterologists were often not confident that these could be sufficient to solve the problem and used macrogol as the first line laxative, both in association with dietetic and lifestyle suggestion and fibers.

Macrogol is effective and safe, and new liquid formulations make it easier to dose; because taste is an important factor for patients’ adherence, particularly for long-time treatment, the formulations without aroma made it more acceptable to patients [27]. On the other hand further increasing fibers intake could induce bloating and abdominal discomfort without improving colonic transit time [28]. To control the different symptoms of IBS (mainly abdominal pain and bloating) gastroenterologists also used antispasmodic drugs, psychotherapy and anti-bloating agents, whereas pelvic floor rehabilitation was suggested more often in FC patients, in whom functional defecation disorders should be more frequent.

Surgery procedures (and sacral neurostimulation) were infrequently suggested by gastroenterologists.

The gastroenterologists involved in this study rarely prescribed laxatives such as lactulose/lactitole, and stimulant, emollient or saline laxatives which still represent the most used laxatives in Italy. These drugs, which cover about 40% of the Italian market [29], are more often prescribed by general practitioners [23] and other specialists than gastroenterologists.

Prucalopride, recently available on the Italian market, was prescribed in about 13% of patients although it was considered, probably because expensive, a second/third line treatment, and prescribed more frequently at a follow-up. At the time of the study, linaclotide was not yet available on the Italian market.

As previously reported for diagnostic tools, the amount of therapy prescribed also increased by increasing PAC-SYM and PAC-QoL scores; in NRC patients, who displayed lighter symptoms, fewer therapies were suggested. In conclusion, in our country a gastroenterological evaluation of CC is often delayed in patients with long lasting symptoms, colonoscopy and blood tests are considered a “first line” diagnostic tool, and DRE is insufficiently performed. Furthermore, constipation is associated with several comorbidities in most patients. Among Italian gastroenterologists macrogol is the most frequently used laxative, while in IBS-C patients a larger amount of drugs is prescribed than in FC and NRC patients.

The study also provides several educational ideas to improve the diagnostic and therapeutic approach to CC: general practitioners and other specialists should be suggested to address earlier such patients to a gastroenterologist before long-term complications occur. DRE should be performed in all patients, while echography usefulness should be resized.

## Conclusions

Chronic constipation is a common disorder that has a remarkable impact on the quality of life. We report on diagnostic and therapeutical experiences of Italian gastroenterologists.

Patients with irritable bowel syndrome with constipation reported more severe symptoms and worsened quality of life than functional constipation. Colonoscopy and blood tests were the most prescribed tests and Macrogol was the most prescribed laxative.

This study can provide several educational ideas to improve the diagnostic and therapeutic approach to Chronic Constipation.

## Appendix

### ChroCoDiTE Study Group Members - Collaborators

**Table:**

Last Name, First Name, Degree	Affiliation
Bellini Massimo, MD	U.O. Gastroenterologia Universitaria – AOU Pisana, Pisa
Usai Satta Paolo, MD	S.C. Gastroenterologia - Azienda Ospedaliera G. Brotzu - Cagliari
Bove Antonio, MD	U.O. Gastroenterologia ed Endoscopia Digestiva, Dipartimento di Gastroenterologia - AORN "A. Cardarelli”, Napoli
Bocchini Renato, MD	Fisiopatologia ed Endoscopia Digestiva. Gastroenterologia ed Endoscopia Digestiva, Casa di Cura Malatesta Novello, Cesena
Battaglia Edda, MD	S.O.C Gastroenterologia, Ospedale Cardinal Massaja, Asti
Alduini Pietro, MD	U.O. Gastroenterologia; Ospedale di Lucca
Galeazzi Francesca, MD	U.O. Gastroenterologia Universitaria - Azienda Ospedaliero Universitaria Padova
Bassotti Gabrio, MD	Sezione di Gastroenterologia ed Epatologia, Dipartimento di Medicina Interna, Università di Perugia
Balzano Antonio, MD	Azienda Ospedaliera di Rilevanza Nazionale "A. Cardarelli”, Napoli
Portincasa Piero, MD	Clinica Medica "A. Murri", Dipartimento di Scienze Biomediche e Oncologia Umana, Università di Bari
Bonfrate Leonilde, MD	Clinica Medica "A. Murri", Dipartimento di Scienze Biomediche e Oncologia Umana, Università di Bari
D’Alba Lucia; MD	U.O.C. Gastroenterologia ed Endoscopia Digestiva, Az. Ospedaliera San Giovanni Addolorata - Roma
Badiali Danilo, MD	Dip. Medicina Interna e Specialità Mediche, La Sapienza, Roma
Marchi Santino, MD	U.O. Gastroenterologia Universitaria – AOU Pisana, Pisa
Gambaccini Dario, MD	U.O. Gastroenterologia Universitaria – AOU Pisana, Pisa
Neri Maria Cristina, MD	Ambulatorio Gastroenterologia ed Endoscopia digestiva – Pio Albergo Trivulzio, Milano,
Muscatiello Nicola, MD	U.O. Gastroenterologia Universitaria, Foggia
Di Stefano Michele, MD	U.O. Medicina Interna I, Fondazione IRCCS Policlinico "S.Matteo", Pavia.
Giannelli Claudio, MD	U.O.C. di Gastroenterologia Riabilitativa – Az. Ospedaliera San Camillo – Forlanini, ROMA
Goffredo Fabio, MD	U.O.C di Gastroenterologia Riabilitativa – Az. Ospedaliera San Camillo – Forlanini, ROMA
Turco Luigi, MD	S. S. Endoscopia Digestiva; U. O. C. Chirurgia Generale; P. O. Copertino, Lecce
Camilleri Salvatore, MD	U.O.C. di gastroenterologia, Ospedale M. Raimondi San Cataldo, Caltanissetta
Ceccarelli Giovanni, MD	U.O.C. di Gastroenterologia ed Endoscopia Digestiva, Ospedale della Versilia - Viareggio
Iovino Paola, MD	Dipartimento di Medicina e Chirurgia-Università di Salerno
Montalbano Luigi Maria, MD	U.O.C. di Gastroenterologia, Ospedali Riuniti Villa Sofia-Cervello Palermo
Morreale Gaetano Cristian, MD	Gastroenterologia ed Epatologia; Policlinico Palermo
Rentini Silvia, MD	U.O.C. Gastroenterologia ed Endoscopia Operativa, Dipartimento Oncologico; Azienda Ospedaliera Universitaria Senese - Siena
Savarino Vincenzo, MD	U.O. Clinica Gastroenterologia con Endoscopia - IRCCS AO Universitaria San Martino - IST - Genova
Segato Sergio, MD	UO Gastroenterologia ed Endoscopia Digestiva. AOU Macchi, Varese
Buscarini Elisabetta, MD	U.O. Gastroenterologia ed Endoscopia Digestiva, Ospedale Maggiore- Crema
Manfredi Guido, MD	U.O. Gastroenterologia ed Endoscopia Digestiva, Ospedale Maggiore- Crema
Cannizzaro Renato, MD	SOC. Gastroenterologia Oncologica, Centro di Riferimento Oncologico - Istituto Nazionale Tumori IRCCS, Aviano
Passaretti Sandro, MD	U.O. Gastroenterologia Ospedale Universitario San Raffaele, Milano, Italy
Alessandri Matteo, MD	U.O. Gastroenterologia Ospedale Universitario San Raffaele, Milano, Italy
Corti Federico, MD	U.O.C. di Gastroenterologia ed Endoscopia Digestiva, Ospedale della Versilia - Viareggio
Cuomo, Rosario, MD	Dipartimento di Medicina Clinica e Sperimentale, Università Federico II, Napoli
Zito Francesco Paolo, MD	Dipartimento di Medicina Clinica e Sperimentale, Università Federico II, Napoli
Mellone Carmine, MD	UOS di Endoscopia Digestiva. Ospedali della Valdichiana, Montepulciano
Barbera Roberta, MD	Ospedale San Giuseppe Multimedica - Milano
Milazzo Giuseppe, MD	UOC Medicina e Lungodegenza, Ospedale Vittorio Emanuele III, Salemi
Pucciani Filippo, MD	Dipartimento di Chirurgia e Medicina Traslazionale - Università di Firenze.
Soncini Marco, MD	UOC Gastroenterologia, Ospedale San Carlo - Milano
Lai Maria Antonia, MD	Dipartimento di Medicina Interna A.O.U. Policlinico di Monserrato, Università di Cagliari
Ruggeri Maurizio, MD	U.O. Gastroenterologia, Ospedale Sant’Andrea - Roma
Savarese Maria Flavia, MD	U.O.C. Endoscopia Digestiva e Gastroenterologia – A.O. Istituti Ospitalieri di Cremona
De Bona Manuela, MD	U.O.C. Gastroenterologia, Ospedale S. Maria Del Prato, Feltre
Surrenti Elisabetta, MD	SOS Fisiopatologia dell'apparato digerente e motilità, AOU Careggi - Firenze
Arini Andrea, MD	U.O. Gastroenterologia ed Epatologia; Policlinico Paolo Giaccone - Palermo
Dinelli Marco, MD	U.O. Endoscopia Digestiva, Azienda Ospedaliera San Gerardo, Monza
Leandro Gioacchino, MD	Dipartimento di gastroenterologia, IRCCS De Bellis – Castellana Grotte
Peralta Sergio, MD	U.O.C di Gastroenterologia ed Epatologia, A.O.U. Policlinico Paolo Giaccone - Palermo
Manta Raffaele, MD	U.O.C. Endoscopia Digestiva ed Interventistica Ospedale Niguarda Ca' Granda , Milano
Quartini Mariano, MD	S.C. Epatologia e Gastroenterologia, A.O. S. Maria, Terni
Torresan Francesco, MD	Dipartimento di Medicina Interna e Gastroenterologia , Policlinico S.Orsola-Malpighi - Università di Bologna,
Vilardo Luigi, MD	U.O.S. Gastroenterologia Ospedale Generale Ferrari, Castrovillari
Pulvirenti D’Urso Antonino, MD	U.O. Chirurgia, Ospedale Nuovo Garibaldi - Catania
Tarantino Ottaviano, MD	U.O. Gastroenterologia, Ospedale S. Giuseppe, Empoli
Noris Roberto Antonio, MD	U.O.C. di Gastroenterologia ed Endoscopia Digestiva - A.O. Bolognini - Seriate
Monica Fabio, MD	S.C. Gastroenterologia ed Endoscopia Digestiva – AOU - Ospedale di Cattinara - Trieste
Carrara Maurizio, MD	U.O.S.D. di Gastroenterologia Ospedale Orlandi, Bussolengo
Losco Alessandra, MD	Gastroenterologia Ospedale San Paolo - MILANO
Lauri Adriano, MD	U.O.C. Gastroenterologia ed Endoscopia Digestiva, Ospedale Civile Spirito Santo, Pescara
Neri Matteo, MD	Dipartimento di Medicina e Scienze dell'Invecchiamento & Ce.S.I., Università G. D'Annunzio, Chieti.
Grassini Mario, MD	S.O.C. Gastroenterologia, Ospedale Cardinal Massaja, Asti

## Additional file

Additional file 1: Data: the PAC-SYM and PAC-QoL total and domain scores. (DOC 17 kb)

## Abbreviations

AIGO: Italian association of hospital gastroenterologists and endoscopists
BMI: Body mass index
CC: Chronic constipation
DRE: Digital rectal examination
FC: Functional constipation
IBS-C: Irritable bowel syndrome with constipation
NRC: No Rome constipation
PAC-QoL: The patient assessment of constipation-quality of life
PAC-SYM: Patient assessment of constipation-symptoms
QoL: Quality of life.
