# Supplementary material for: Chronic constipation diagnosis and treatment evaluation: the “CHRO.CO.DI.T.E.” study
Source: BMC Gastroenterol. 2017 Jan 14;17:11. doi: 10.1186/s12876-016-0556-7 (PMC5237544; doi:10.1186/s12876-016-0556-7)
Supplement: Additional file 1: — Data: the PAC-SYM and PAC-QoL total and domain scores. (DOC 17 kb) [file 12876_2016_556_MOESM1_ESM.doc]

APPENDIX A

PAC-SYM overall score = (sum of scores of all non-missing items) (number of non-missing items) if at least 6 items are non-missing; value is missing if more than 6 items are missing.

Subscale values are defined as (sum of scores of non-missing included items) divided by (number of non-missing included items) if the missing data handling rule is not met. The value is missing if the missing data handling rule is met.

The table below defines the included items and missing data handling rules for each subscale.

|  | **Included items** | **Missing data handling rule** |
| --- | --- | --- |
| PAC-SYM abdominal symptoms | 1-4 | If >2 items are missing, set scale to missing. |
| PAC-SYM rectal symptoms | 5-7 | If >1 item is missing, set scale to missing. |
| PAC-SYM stool symptoms | 8-12 | If >2 items are missing, set scale to missing. |

APPENDIX B

PAC-QoL overall score = (sum of scores of all non-missing items) / (number of non-missing items) if at least 14 items are not missing; value is missing if more than 14 items are missing.

Subscale values are defined as (sum of scores of non-missing included items) divided by (number of non-missing included items) if the missing data handling rule is not met. The value is missing if the missing data handling rule is met.

The table below defines the included items and missing data handling rules for each subscale.

|  | **Included items** | **Missing data handling rule** |
| --- | --- | --- |
| PAC-QoL Physical Discomfort | 1-4 | If >2 items are missing, set scale to missing. |
| PAC-QoL Psychosocial Discomfort | 5-12 | If >4 items are missing, set scale to missing. |
| PAC-QoL Worries and concerns | 13-23 | If >5 items are missing, set scale to missing. |
| PAC-QoL Satisfaction | 24-28 | If >2 items are missing, set scale to missing. |
